# Draft Genome Sequence of *Exiguobacterium* sp. HVEsp1, a Thermophilic Bacterium Isolated from a Deep-Sea Hydrothermal Vent in the Okinawa Trough

**DOI:** 10.1128/genomeA.00253-17

**Published:** 2017-04-27

**Authors:** Chen Chen, Li Sun

**Affiliations:** a Key Laboratory of Experimental Marine Biology, Institute of Oceanology, Chinese Academy of Sciences, Qingdao, China; b Laboratory for Marine Biology and Biotechnology, Qingdao National Laboratory for Marine Science and Technology, Qingdao, China; c University of Chinese Academy of Sciences, Beijing, China

## Abstract

We report here the draft genome sequence of *Exiguobacterium* sp. HVEsp1, a thermophilic bacterium isolated from a deep-sea hydrothermal vent. The estimated genome size of this strain is 2,838,499 bp with a G+C content of 48.2%. The genome sequence data provide valuable information that will facilitate studies on the adaptation mechanisms of bacteria living in deep-sea hydrothermal vents.

## GENOME ANNOUNCEMENT

The genus *Exiguobacterium*, which was first described in 1983, belongs to the *Bacillales* family XII *incertae sedis*, *Firmicutes* (1). Members of this genus are widespread in markedly diverse habitats, including cold environments such as Siberian permafrost and Antarctic ice, hot/hyperalkaline springs, freshwater, and marine waters (2–6). To date, however, no isolates of the genus *Exiguobacterium* from deep-sea live hydrothermal vent have been sequenced.

*Exiguobacterium* sp. HVEsp1 was isolated from the sediment of an active hydrothermal vent in the Okinawa Trough (27°33′N, 126°58′E; 1391.9 m depth) during a scientific cruise conducted by the scientific research vessel KEXUE in April 2014. Genome DNA was extracted from HVEsp1 using a TIANamp bacteria DNA kit (Tiangen, Beijing, China) according to the manufacturer’s instructions. The genome of HVEsp1 was sequenced by Novogene Bioinformtics Technology (Beijing, China) with the Illumina HiSeq and MiSeq platforms using a combination of short-insert (474 bp) and long-insert (4,959 bp) DNA libraries. Resulting sequences were assembled with SOAPdenovo version 2.04 (7, 8). Putative coding sequences were identified with GeneMarkS version 4.66 (9).

The genome of HVEsp1 is a circular chromosome of 2,838,499 bp with an average G+C content of 48.2%. A total of 11 contigs ranging from 240 bp to 1,561,357 bp (the *N*_50_ and *N*_90_ contig sizes were 1,561,357 bp and 315,782 bp, respectively) were obtained and combined into two scaffolds with lengths of 2,847,549 bp and 1,270 bp (the *N*_50_ and *N*_90_ scaffold sizes were both 2,847,549 bp). The coding region accounts for 89.66% of the chromosome and comprises 2,948 predicted coding sequences (CDSs), with even distribution between the forward (48.03%) and reverse (51.97%) chromosome strands. The 2,948 CDSs comprise 2,862 (97.1%) protein-coding sequences. There are 63 tRNA genes and 23 rRNA genes, the latter being clustered into nine operons, which is common in most species of the genus *Exiguobacterium*. The nine operons are evenly distributed between the forward chromosome strand (four operons) and the reverse strand (five operons). Also present in the HVEsp1 genome are 114 transposons and 40 tandem repeat regions, accounting for ~0.43% of the genome; these elements are known to be involved in genomic rearrangements and horizontal gene transfer in bacteria. Thirteen gene islands were predicted in the genome of HVEsp1, comprising a total of 181,452 bp and 192 predicted CDSs. Of the 2,948 genes, 2,807 (95.2%) were assigned predicted biological functions, with 667 (23.8%) showing similarities to hypothetical proteins from other organisms.

### Accession number(s).

This whole-genome shotgun project has been deposited at DDBJ/ENA/GenBank under the accession number MTCT00000000. The version described in this paper is the first version, MTCT01000000.
